# Determination of the State of Strain of Large Floating Covers Using Unmanned Aerial Vehicle (UAV) Aided Photogrammetry

**DOI:** 10.3390/s17081731

**Published:** 2017-07-28

**Authors:** Wern Hann Ong, Wing Kong Chiu, Thomas Kuen, Jayantha Kodikara

**Affiliations:** 1 Department of Mechanical & Aerospace Engineering, Monash University, Clayton, Victoria 3800, Australia; wing.kong.chiu@monash.edu; 2 Melbourne Water, 990 La Trobe Street, Docklands, Victoria 3008, Australia; Thomas.Kuen@melbournewater.com.au;; 3 Department of Civil Engineering, Monash University, Clayton, Victoria 3800, Australia; jayantha.kodikara@monash.edu

**Keywords:** Unmanned aerial vehicle (UAV), membrane, strain measurement, 3D scanning, photogrammetry

## Abstract

Floating covers used in waste water treatment plants are one of the many structures formed with membrane materials. These structures are usually large and can spread over an area measuring 470 m × 170 m. The aim of this paper is to describe recent work to develop an innovative and effective approach for structural health monitoring (SHM) of such large membrane-like infrastructure. This paper will propose a potentially cost-effective non-contact approach for full-field strain and stress mapping using an unmanned aerial vehicle (UAV) mounted with a digital camera and a global positioning system (GPS) tracker. The aim is to use the images acquired by the UAV to define the geometry of the floating cover using photogrammetry. In this manner, any changes in the geometry of the floating cover due to forces acting beneath resulting from its deployment and usage can be determined. The time-scale for these changes is in terms of weeks and months. The change in the geometry can be implemented as input conditions to a finite element model (FEM) for stress prediction. This will facilitate the determination of the state of distress of the floating cover. This paper investigates the possibility of using data recorded from a UAV to predict the strain level and assess the health of such structures. An investigation was first conducted on a laboratory sized membrane structure instrumented with strain gauges for comparison against strains, which were computed from 3D scans of the membrane geometry. Upon validating the technique in the laboratory, it was applied to a more realistic scenario: an outdoor test membrane structure and capable UAV were constructed to see if the shape of the membrane could be computed. The membrane displacements were then used to calculate the membrane stress and strain, state demonstrating a new way to perform structural health monitoring on membrane structures.

## 1. Introduction

Large membrane-like covers are used in several environmentally sensitive application contexts, including:

1. Floating covers for clean water reservoirs, to prevent evaporation and pollution
2. Landfill covers to stop leakage of hazardous chemicals or harmful matter
3. Mining applications such as heap leaching, salt evaporation ponds and tailings impoundment [1]

These are in addition to their use as floating covers for anaerobic reactors in sewage treatment plants, which has been the motivation for the work presented in this paper. An unexpected failure of these membranes can have severe detrimental effects on the operation, environmental and economic cost. The economic cost is extreme because the cover replacement cost is in the order of tens of millions (~$50 m). In addition, there is also the economic loss associated with the inability to harvest biogas. The cover failure can adversely impact on the operation of the waste treatment plant. It can also result in unpleasant odors being released into surrounding residential areas affecting the air quality. In this respect, early warning of defects, risk or harm to the integrity of the covers is crucial. Whilst the primary aim of these early warning systems can be used to assist with scheduling of repairs or rectification of the situation under the cover, it can also be used to recommend operational changes at the plant to prevent catastrophic failure. In this respect, a robust structural health monitoring methodology will provide timely warnings that are crucial from the point of view of safe continued operation, failure prevention and maintenance management.

**Figure 1:**
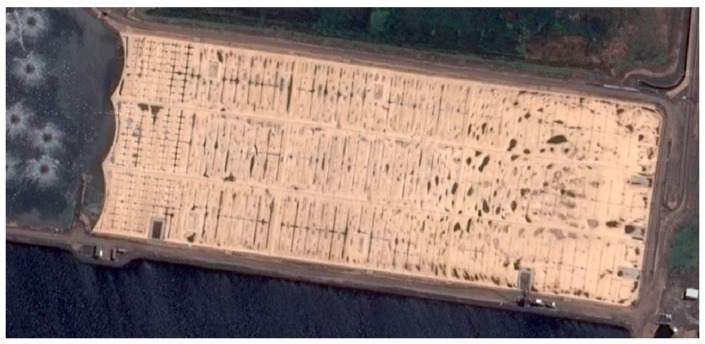
Aerial view of floating cover at Melbourne Water’s Western Treatment Plant.

**Figure 2:**
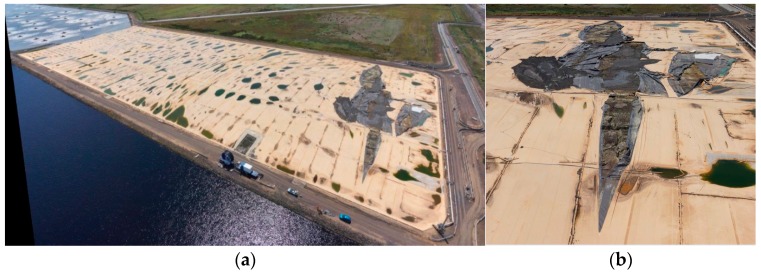
(**a**) Photograph of a failed floating cover, following an unexpected failure in December 2014, exposing the underlying scum-berg; and (**b**) close-up view of the torn cover exposing the underlying scum-berg.

The floating covers used in Melbourne Water’s Western Treatment Plant (WTP) in Werribee, Victoria, were made from an upper layer of 1.14 mm reinforced flexible polypropylene (fPP-R); a 12 mm thick intermediate PE foam and a 0.75 mm underlying HDPE membrane spanning an area of 470 m × 170 m, as illustrated in Figure 1. The cover is held down around its perimeter by clamping strips and mechanical fasteners to provide an airtight seal. All sewage inflow is unscreened and passes first through an anaerobic reactor. As the raw sewage undergoes anaerobic digestion, biogases are produced, which are trapped below the floating cover and harvested for electricity generation. Consequently, there is a high premium placed on ensuring that no failures occur in service. The current maintenance practice involves a visual walk-around inspection which is potentially hazardous and time-consuming, but, more importantly, does not provide advance warning of possible failures, or clear indications of distress in the covers. Indeed, a powerful incentive for the work presented in this paper is the recent catastrophic failure of a cover sheet at the Melbourne Water treatment plant in December 2014, as shown in Figure 2.

A review by Rowe and Sangam [2] shows that HDPE geomembranes are very durable and can have expected service life of over 300 years at 20 °C, and over 45 years at 40 °C [2]. Consequently, well-designed HDPE geomembranes should have long trouble-free lifetimes. During the operation of the reactor, solidified sewage matter can accumulate on the surface of the reactor to form scum-bergs which press against and lift the covers. This deformation has a length scale of around one meter in the vertical direction (uplift). Under conditions of wind loading, the scum-bergs can be displaced laterally, which causes changes to the mechanical stress on the covers. Figure 2a shows an aerial view of the cover after failure. A close-up view of the torn cover is shown in Figure 2b. The structural health management of the floating cover can potentially mitigate the risk of failure. Given that the floating cover also serves to harvest the bio-gas for power generation, a structural integrity based management of the health of the cover can also be ensure the safe operation and continuous delivery of bio-gas. The state of deformation and the corresponding change in the state of strain on the floating cover is a useful indication of the level of distress in the floating cover. These are useful tools for the ongoing health monitoring and management of these large floating covers. In this context, the principal aim of the work in this paper is to correctly monitor the strains and stresses developed in the floating cover due to the uplift associated with these scum-bergs, and to assess the likelihood of failure. This monitoring scheme can also be used as part of the management protocol for the safe operation of the asset in delivering the biogas for power generation. The size of the floating cover and the vast multitude of potential failure locations makes an efficient, non-contact means of integrity assessment extremely attractive. This paper will explore the use of a UAV-aided photogrammetry to determine the profile of the floating cover. This information will be incorporated with a finite element model of the floating cover for assessing the changes in the state of distress of the floating cover.

The development of full-field strain measurement is a field of study that has received significant attention. These works ranges from in-plane strain measurements and those that measure out-of-plane deformation. The works that predominantly reported on in-plane static strain measurement included mature image based non-contact strain measurement technologies. These are currently available in the form of digital image correlation (DIC) and have been reported on by many authors. Grytten et al. [3] used DIC to obtain the full strain field of a ductile thermoplastic specimen which would later be used to measure the stress-strain curve up 100% strain. Similarly, Salvini et al. [4] successfully demonstrated large strains (up to 0.4/40%) could be measured from a polyethylene structure with a painted speckle pattern. While this is beyond what most strain gauges can cope with it was reported that the speckle pattern tends to degrade at such strain levels. This point is reiterated by Iadicola [5] who investigated uncertainties in DIC at large strains. It was reported that the uncertainty in strain increased two orders of magnitude when the specimen went from 0.02% to 50% engineering strain. Another challenge of using DIC with large deformation structures is decorrelation. Pan et al. [6] addressed this by using incremental DIC calculations provided sufficient images have been recorded.

The works reported on the measurement of out-of-plane deformation are often associated with the need for defining the dynamic response of large membrane structures. Indeed, the ability to predict the strain field from 3D information is a challenge. However, a very good example of this work is recently described by Baqersad et al. [7] where a very comprehensive description of the work devoted to the integration of 3D structural deformation with finite element analysis (FEA) for full field strain prediction. In their work, the 3D deformation of the structure (wind turbine blade) is defined by the movement of the reflective dots attached to the structures. In the works reported by Baqersad et al. [8,9,10], they integrated photogrammetry output describing the 3D dynamic response of a wind-turbine blade with finite element analyses to predict the dynamic strain experienced by the blade. It is noted that the strain measured were in the order of 300 microstrains.

Majority of the work presented above utilize two fixed cameras. They also require a pattern to be imposed on the structure. The work presented in this paper is conducted using a single camera mounted on an unmanned aerial vehicle (UAV). The location of the camera is defined by the GPS on the UAV. The 3D feature of the membrane can be determined from an array of photos taken from the camera using a pre-determined flight path. Details of this will be presented in this paper.

In this respect, the work presented in this paper describes an alternative methodology using the 3D photogrammetry as inputs to determine the full field strain measurements on membrane structures. In our work, the membrane structure is essentially static. The time-scale associated with the causes giving rise to the vertical deformation of the cover is in terms of weeks or months. Two of the significant challenges associated with the monitoring of strain on the membrane structure is that it is totally exposed to the environment and is expected to have a life-span of 20 years. It is exposed to direct sunlight and driving rain every day of the year. It is therefore impractical to “seed” the membrane with reflecting markers. In this regard, we seek to assess the viability of using a direct measurement of the deformation of the membrane to predict the state of strain in the cover.

It is expected that the scum-berg formed beneath the floating cover will impose a vertical displacement in the region where it is formed and accumulated. We seek to utilize a methodology that employs 3D scanning to digitize the membrane geometry; the elevation is then used provide the loading condition in terms of imposed displacement on a numerical model which will return the strain field. Interestingly the introduction of the 3D shape as the loading condition is consistent with that reported by Zhao et al. [11]. The current work presented will focus on the development of this capability to predict the state of strain arising from a vertical protuberance. The paper begins with a laboratory study where a small scale membrane structure was instrumented with strain gauges. The strain gauges provided direct strain measurements which could be compared against the non-contact strain measurements to confirm the validity of the methods proposed within this paper. It is evident that the change in the state of the strain field on the membrane structure can be obtained. Knowledge of the boundary conditions will be required to fully define the absolute strain field (Zhao et al. [11]). Upon validating the methodology, the same methods were applied to a large scale roof top membrane confirming the feasibility of applying non-contact strain measurement on large scale membranes.

## 2. Approach to Monitoring Membrane Structures

The aim of the work presented is to facilitate the development of a UAV-aided photogrammetry to quantify the change in the state of strain on the membrane structures resulting from changes in the geometry of the membrane due to a vertical displacement. The work described in this paper demonstrates the potential of using changes in spatial geometry of a membrane structure to ascertain its state of strain via finite element modeling. The size of the membrane considered called for a capability that can define the spatial geometry of a large structure. In the above works, the subject measured are “seeded” to enable the definition of the deformed shape. Pappa et al. [12], did some interesting work comparison on the use of a variety of techniques (reflective markers and projected dots) to define the dynamic motion of membrane. The challenges posed by these techniques are detailed in their paper. Our current work investigated into the potential of using ambient features on the membrane to assist with the 3D reconstruction of the membrane structure and to integrate this into a finite element model to estimate its state of stress and strain.

When considering available remote sensing options for UAVs photogrammetry and LIDAR are readily available. Photogrammetry uses a series of photos and image recognition algorithms to compute the 3D shape of a structure while LIDAR utilizes an array of laser range finders determine the 3D location of millions of points lying on the target structure. LIDAR has the key advantage of better penetration than photogrammetry; this is advantageous where foliage and plants may obscure the ground. However, this penetration is not required for the floating cover because it is a planar structure which only requires surface observations. It is also less susceptible to artifacts and computational errors because the placement of LIDAR points is straight forward compared to image recognition. Consequentially, photogrammetry requires computationally intensive post processing to construct 3D point clouds while LIDAR is able to construct them in real time. Both technologies are at a mature state where they can produce accurate 3D point clouds which are identical. However, photogrammetry has a much lower cost and the ability to capture textures allows tracking of points which may be used for stress analysis by digital image correlation in future. For these reasons, photogrammetry was chosen. Baqersad et al. [7] provided a very comprehensive review of photogrammetry and optical methods for structural dynamics. Given the time-scale associated with our problem at hand where the development beneath the floating cover takes several weeks or month, it can be considered a static problem.

Considering the expected lifespan of the floating cover (>20 years), integrating sensing elements on the floating cover may not be suitable. It is observed that the failure of the floating cover is associated with the excessive deformation of the floating cover due to the accumulation and movement of the scum-berg. To this end, an understanding of the changing profile of the floating cover as a function of operational months or years can form an integral part of the structural integrity assessment of the floating cover. To achieve this aim, the focus of this paper is to determine the capability of a photogrammetry-based methodology to define the profile of a given terrain feature of similar length scale of the scum-berg found at the WTP.

**Figure 3:**
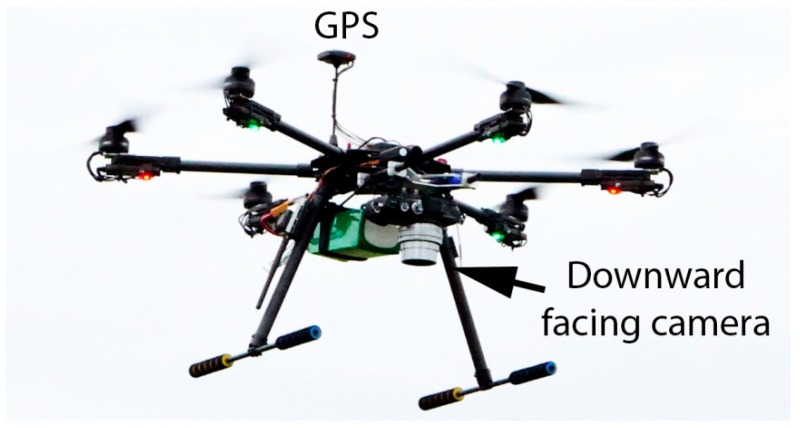
Hex-copter with GPS and downward facing camera.

A bespoke UAV was assembled to perform the photogrammetry tasks which would enable computation of 3D point clouds as shown in Figure 3. The UAV is propelled by a DJI E800-6 propulsion system and powered by a 220 Wh lithium polymer battery. The takeoff weight is approximately 3.5 kg resulting in a usable flight time of around 30 m. An open source Pixhawk flight controller made by 3DR is used to fly the UAV, trigger the camera and log the GPS coordinates for post processing. An Olympus E-PL7 with 14–42 lens was attached in a downward facing orientation to capture the photos which would be used to perform photogrammetry.

**Figure 4:**
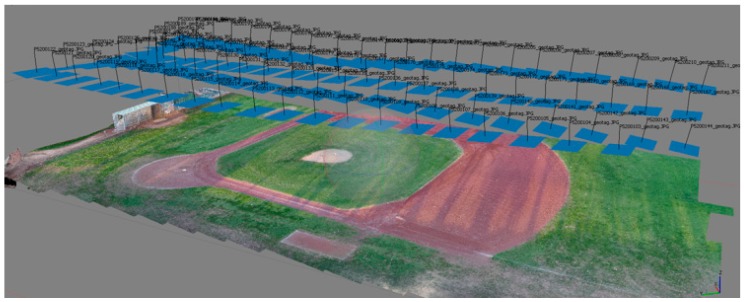
Photo locations taken by the UAV over the baseball pitch

**Figure 5:**
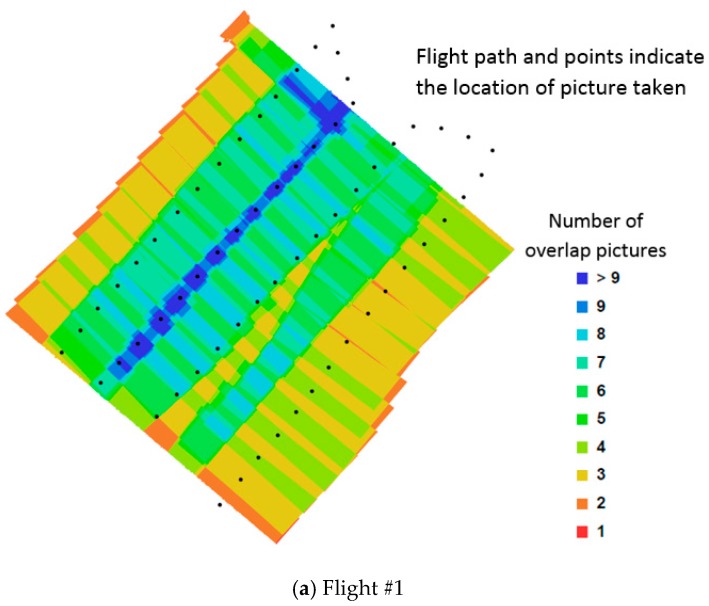

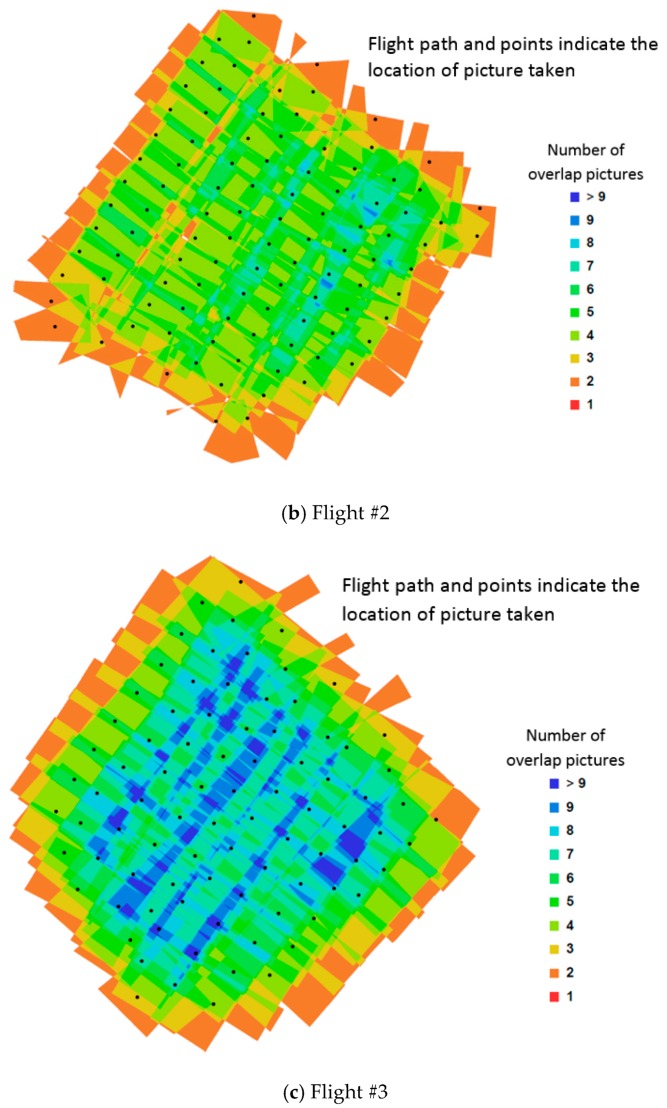
Diagram showing the overlaps of each flight path and the locations where photos were taken.

The issues of radial distortion in 3D reconstruction (Wu [13]) is well documented. Indeed, our work preliminary work (Chiu et al. [14]) was performed to adequately define the parameters required to avoid these issues (see also Li et al. [15]). As reported in Chiu et al. [14], the scanning of a large area (1881 m^2^) took approximately 4 m. This provides an efficient means towards the development of a robust non-contact strain measurement strategy for large membrane. The ability of the photogrammetry methodology to define the details of an undulating terrain was presented by Chiu et al. and is briefly described in this paper. Chiu et al. reported on experiments where the UAV based photogrammetry was tested on a baseball ground, with a pitcher mound length-scale of relevant to the problem at hand. They reported on the average error between the reported GPS location and the photogrammetry estimated position of the feature. This is summarized in Table 1 adapted from Chiu et al. [14]. Figure 4 shows an example of the location of the photos taken by the UAV during flight #1. The flight overlap of each of the flight path is shown in Figure 5. It is evident from this test that a significant overlap is required for an accurate representation of the 3D profile.

**Figure 6:**
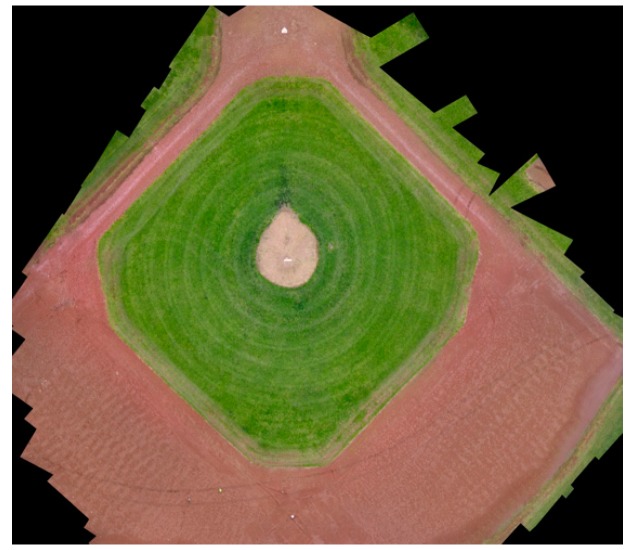
Reconstructed orthophoto from multiple photographs.

**Figure 7:**
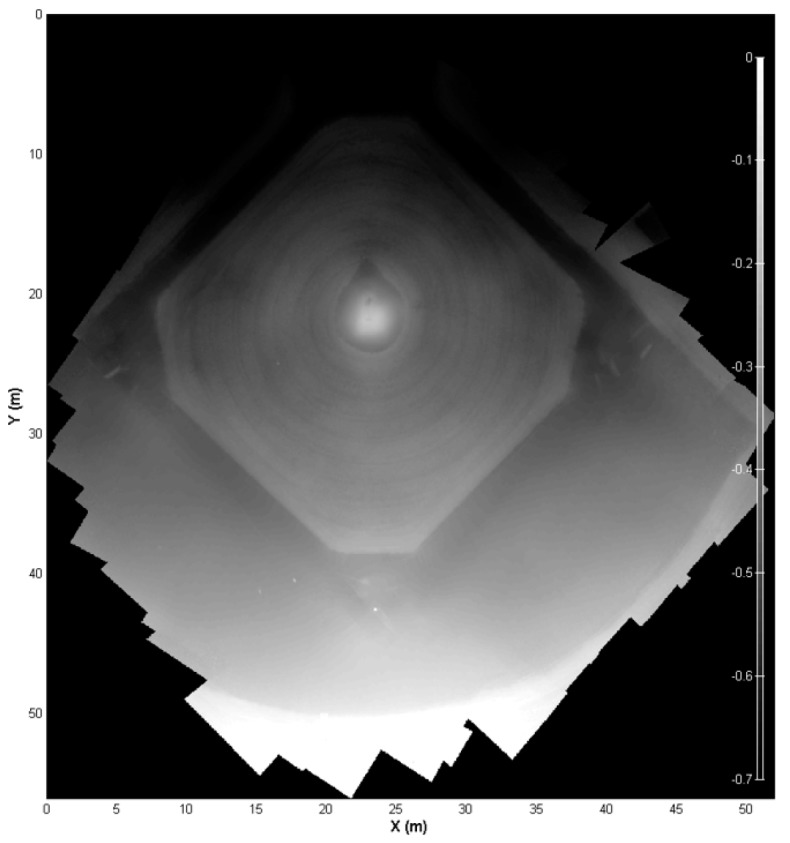
Depth elevation map.

**Figure 8:**
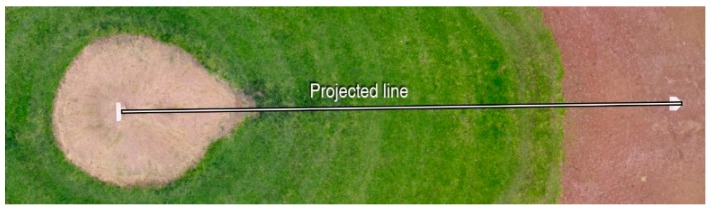
Reconstructed orthophoto from multiple photographs.

**Figure 9:**
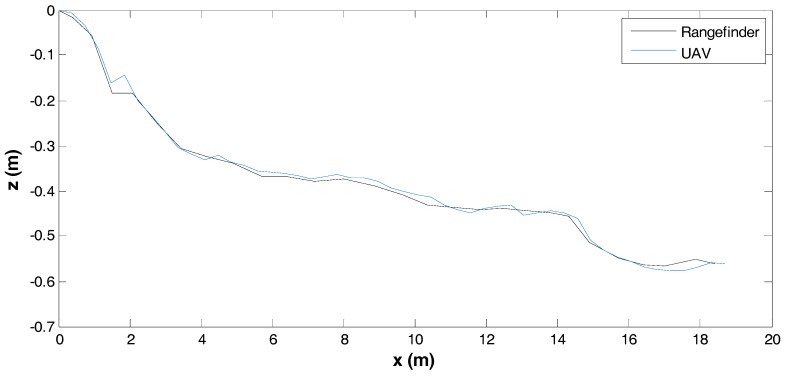
Comparison of elevation measured over the pitcher mound.

Figure 6 shows the orthophoto from flight #3. The elevation map of orthophoto is shown in Figure 7. The elevation of the pitcher mound is measured and compared with the photogrammetry output along a line shown in Figure 8. Figure 9 shows the comparison of these measurements. A good agreement is obtained.

**Figure 10:**
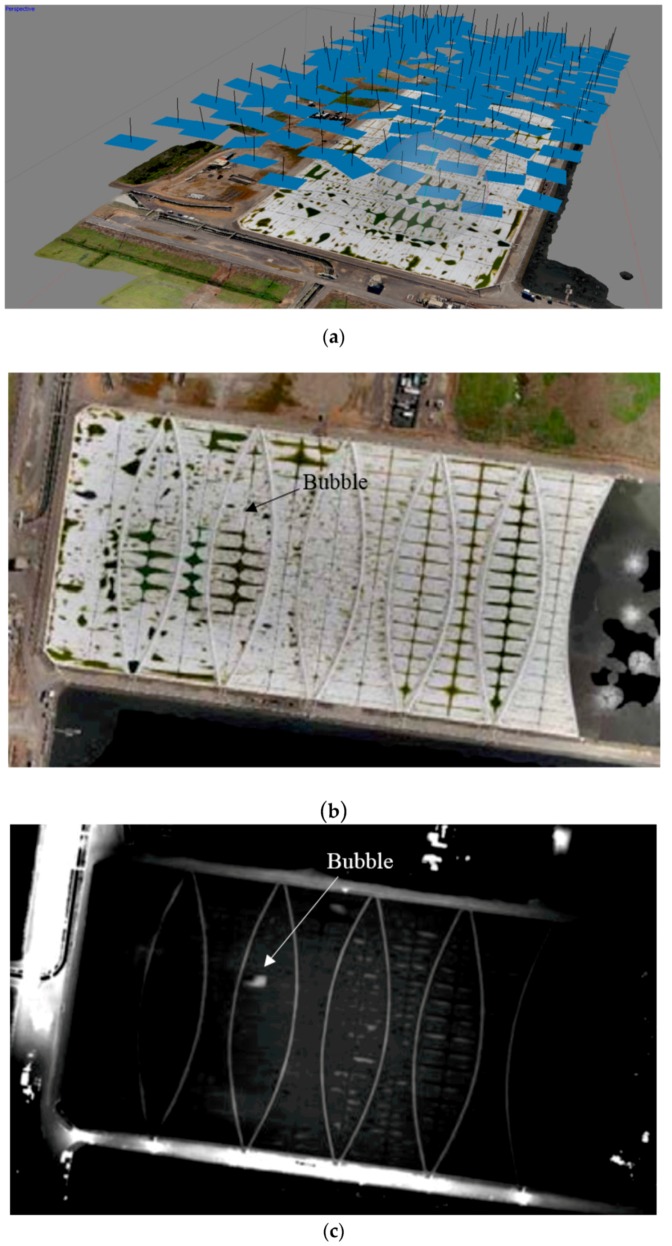
(**a**) Locations where photos were taken (133 photos); (**b**) Orthophoto from images; (**c**) Elevation map of the floating cover.

The photogrammetry capability was subsequently applied to the definition of the floating cover at the Western Treatment Plant. Figure 10a shows the locations where the photos were taken over the floating cover. The orthophotos and the elevation map derived from these photos are shown in Figure 10b,c, respectively. The presence of the “bubble” on the floating cover due to the accumulation of biogas was identified. This result clearly demonstrates that UAV-aided photogrammetry is a valuable and efficient tool for the health monitoring and management of these large floating covers. The challenge is to be able to utilize this information to characterize the change in the state of strain when the geometry changes with usage.

Once the spatial geometry of the membrane is obtained from photogrammetry, it will be used to create a finite element model. Over time, the membrane structure will deform into different shapes due to the formation of scum-bergs. When these scum-bergs appear, new spatial geometry data will be obtained by the UAV. The deformation resulting from the formation of a scum-berg can be characterized by the change in the geometry. These data are then used to load the finite element model to compute the strain field across the membrane structure. This technique is similar to that reported by Zhao et al. [11]. This method of integrity assessment of the floating cover is a new area of work that has attracted considerable interests due to the high cost of replacement and the economic benefits one can derive from the safe operation of the floating cover.

## 3. Definition of Input Conditions for Stress Analyses of Laboratory Membrane Using Photogrammetric Outputs

**Figure 11:**
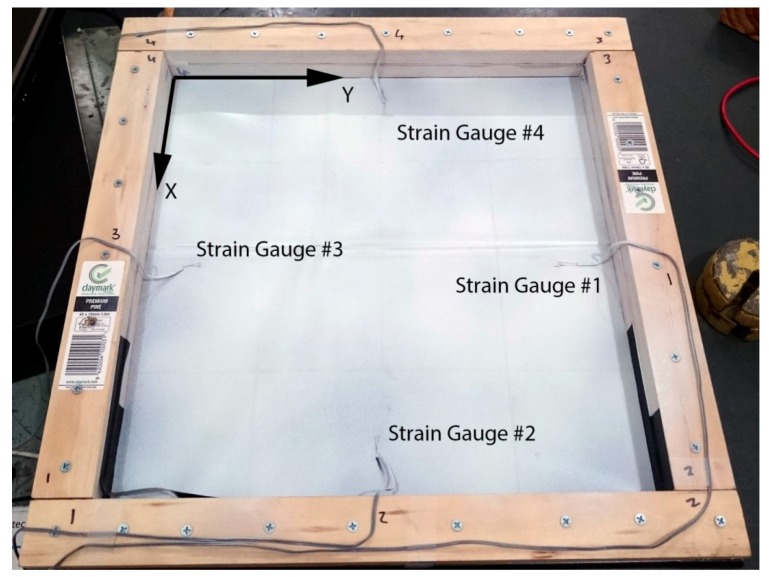
Laboratory membrane experimental setup.

In order to validate the proposed approach, a laboratory study was first conducted. The test structure is shown in Figure 11 consisting of a PVC membrane with an area 400 mm × 400 mm and four bonded strain gauges. During construction, the membrane was laid flat on a table, folded around the wooden frame and clamped with another wooden frame. This means the zero stress state is almost a flat plane since the membrane was supported during installation.

The four unidirectional strain gauges (type FLA-3-350-11-1L, Tokyo Sokki Kenkyujo Co. Ltd.) were bonded onto the test membrane. Each of them is oriented to measure strain towards the middle of the membrane, i.e., Gauges 1 and 3 in the Y direction, and 2 and 4 in the X direction. They will be used to provide direct strain measurements on the membrane which can be compared to strains which were estimated from the scanned deformation.

**Figure 12:**
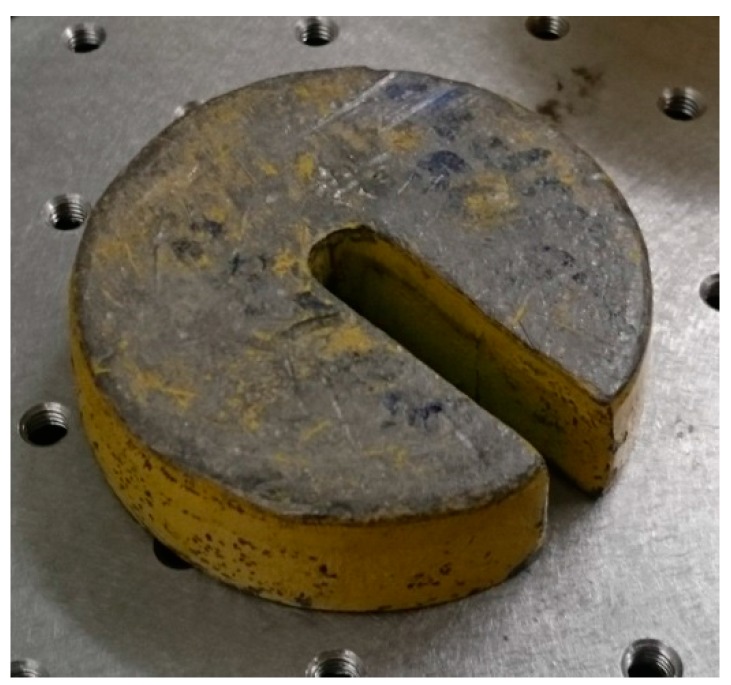
Spacer to be placed beneath the test membrane to simulate the formation of scum-berg. (72 mm diameter by 20 mm thick).

**Figure 13:**
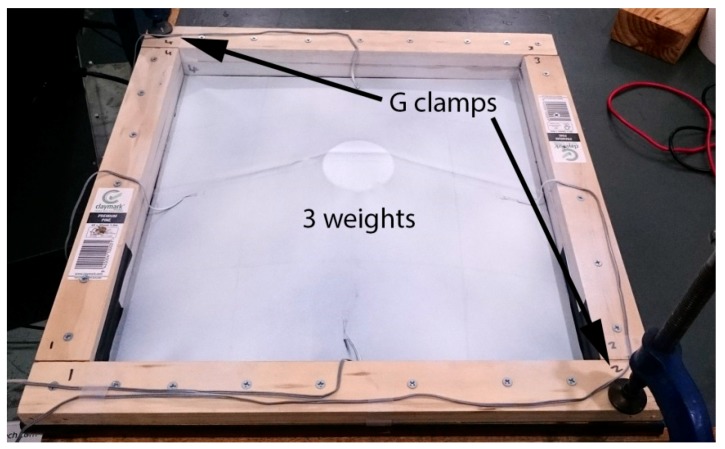
Test membrane with three spacers (simulated scum-berg) placed under the membrane.

The aim of our work is to simulate the formation of the scum-berg beneath the floating cover that will impose a vertical displacement in the region where it is formed and accumulated. We seek to simulate this by placing circular spacers beneath the test membrane. In this respect, during the experiment, circular spacers (Figure 12) were placed under the membrane to create a deformation that is similar to that expected from a scum-berg. The spacers were incrementally added under the membrane as shown in Figure 13 to simulate the development of the scum-berg. A maximum of three spacers were used. The edges of the test rig were fastened to the table using G clamps. This will also help ensure a consistent displacement of the membrane.

**Figure 14:**
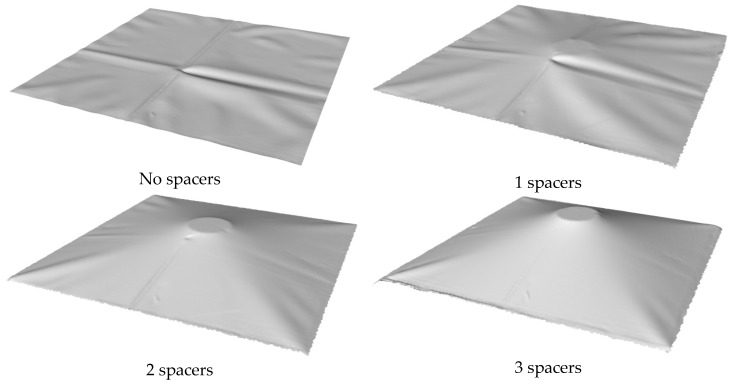
Geometry of the test membrane subjected to vertical deformation arising from the simulated scum-berg obtained from 3D scanning.

The geometry of the test membrane was scanned at every load case and the readings from the strain gauges were recorded. Figure 13 shows the deformed test membrane with three spacers placed beneath. While the rooftop experiment discussed in Section 4 will have its 3D geometry determined by means of UAV and GPS with post processing, the small scale of the laboratory study and lack of GPS reception meant this is not be possible. Instead, this task was achieved by a DAVID SLS-2 commercially available structured light scanning (SLS) system. In order to help the SLS system the membrane was spray painted matte white. Figure 14a–d show the deformed test membrane as scanned by the SLS system.

**Figure 15:**
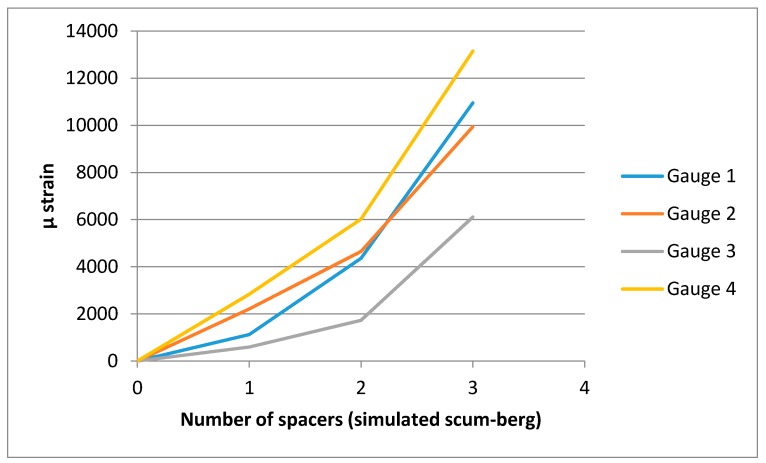
Strain recorded as a function of the simulated scum-berg formation beneath the membrane.

The accompanying strain readings located on the membrane were recorded at a rate of 100Hz upon the application of a spacer. All strain gauges were zeroed at the beginning and then one spacer placed underneath and G clamps tightened. Interestingly, the strain is time dependent and does not settle until approximately 60 s after the load is applied. Table 2 summarizes the strain gauge reading results once they have reached steady state. The last µstrain values were recorded for each stage. A graphical representation of how the strains increase with spacers is also shown in Figure 15. This figure also shows the strain appears to be rising at an increasing rate with each additional spacer. This is expected due to the increasing membrane angle with respect to a flat plane.

**Figure 16:**
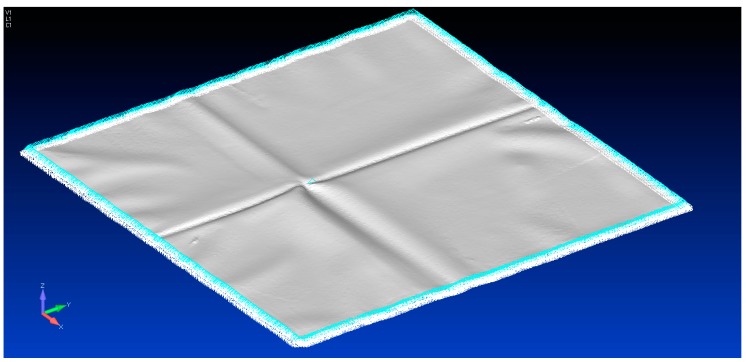
Imported geometry of the test membrane in its unloaded condition with edge constraints imposed.

Using the 3D scanned geometry, a model was built in Femap, as shown by Figure 16. The zero load state was imported as 3D triangular mesh and converted into plate elements. All the nodes at the edge of the plate were given a fixed constraint, as shown in Figure 16. This model has 66,855 elements.

The material properties of the PVC membrane was determined by tensile testing using a test specimen measuring 56 mm by 35 mm excluding clamp area. The tensile test specimen was tested at a displacement rate of 10 mm/min to a maximum deformation of 10 mm. The calculated Young’s modulus of approximately 20 MPa in the 0–0.1 strain region is within the expected range for flexible PVC [16].

Since this is a large deformation problem, the NX Nastran nonlinear solver was used. This means the model was incrementally loaded in steps and the geometric deflections were updated at each step. Plate elements were used instead of membrane elements because membrane elements have no bending stiffness. This creates a divide by zero operation at the beginning when the membrane is mostly flat and an out of plane force is applied making the model unsolvable. The plate elements were assigned a 0.4 mm thickness, as specified by the manufacturer, which provides a non-zero bending stiffness at the beginning allowing the model to be solved. However, when the non-linear model has solved its final load step, bending has negligible effect and the stresses will be dominated by membrane stresses. A script was written to load the model by enforcing the elevation maps onto the model for every node according to the equation;

(1) $$dZ\left(x,y\right)=Z_{Sj}\left(x,y\right)-Z_{S0}\left(x,y\right)$$

where dZ(x,y) is the enforced out of plane displacement for any node, $Z_{Sj}\left(x,y\right)$ is the elevation field for state j where j is the number of spacers, and $Z_{S0}\left(x,y\right)$ is the elevation field for the state with no spacers and zero load. The array dZ(x,y) acts as the input condition for the finite element model of the deformed membrane. This approach has drawn from the work presented by Zhao et al. [11].

**Figure 17:**
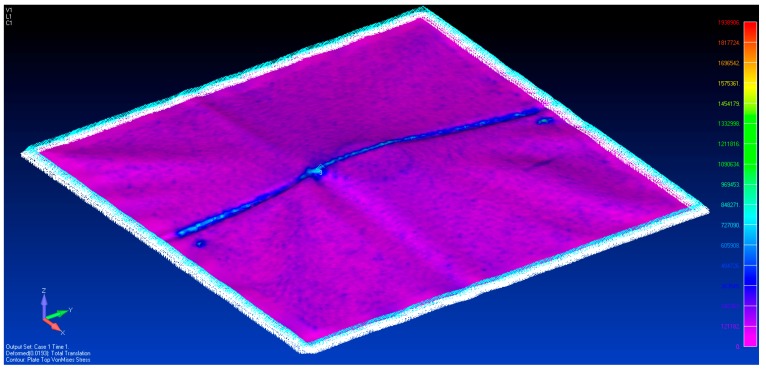
The von Mises stress distribution with one spacer placed beneath the membrane.

**Figure 18:**
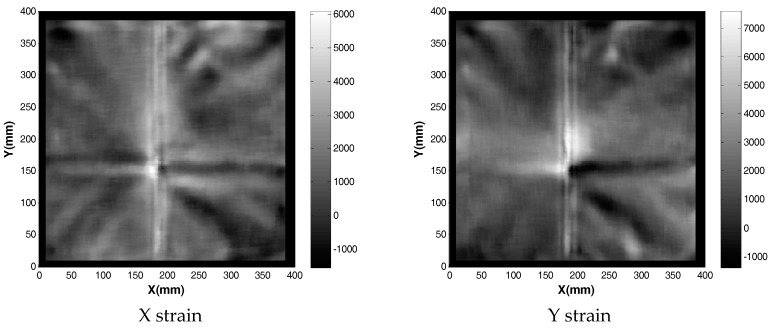
Strain field obtained when membrane was loaded with one spacer added beneath the membrane.

The solution for von Mises stress and deformed shape when one spacer is used is shown in Figure 17. Apart from the localized stresses at the fold line running in the Y axis direction, there appears to be no elevated stress across the membrane from loading it with 1 spacer. This suggests there is a certain deformation threshold that must be exceeded before the non-contact method can detect strain increases. In order to confirm these observations, strain readings were taken from the model. They were exported into MATLAB where a 20 mm median filter was applied to smooth out the high (spatial) frequency localized strain variations. The resulting data are shown in Figure 18.

**Figure 19:**
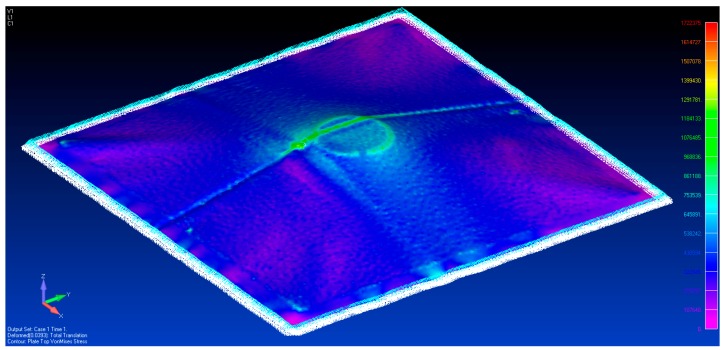
The von Mises stress distribution with two spacers placed beneath the membrane.

**Figure 20:**
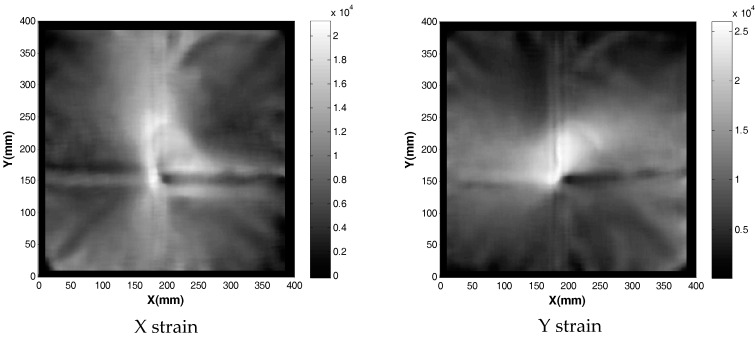
Strain field obtained when membrane was loaded with two spacers added beneath the membrane.

**Figure 21:**
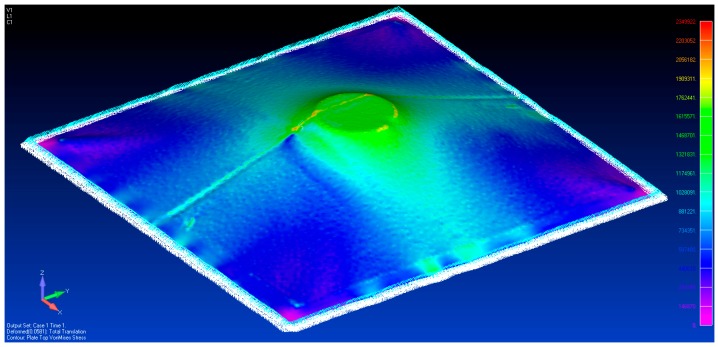
The von Mises stress distribution with three spacers placed beneath the membrane.

**Figure 22:**
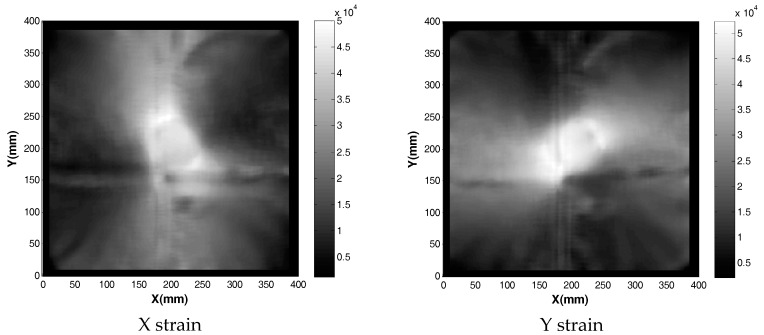
Strain field obtained when membrane was loaded with three spacers beneath the membrane.

**Figure 23:**
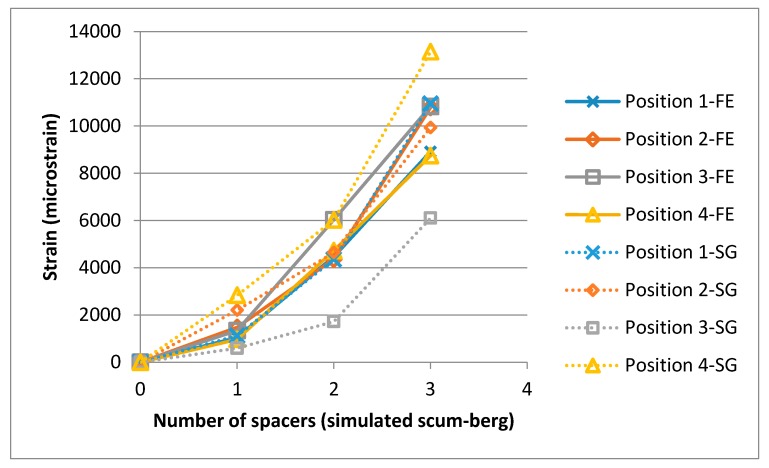
Strain recorded as a function of the simulated scum-berg formation beneath the membrane (FEA and strain gauge (SG)).

The process was repeated for the deformation recorded with two and three spacers under the membrane, resulting in Figure 19, Figure 20, Figure 21 and Figure 22. As the loading is increased with more spacers, the level of stresses and strains become more pronounced, as expected. This leads to an increasing degree of certainty in the predicted stress and strain as the deformations get larger. A summary of the strain readings from these models is presented in Table 3 and visualized in Figure 23 where the gauge locations match those labeled in Figure 11. This makes it possible to compare the non-contact measurement of strains against those measured by strain gauge. The agreement between the measured and predicted strain levels shown in Figure 23 is good. This confirms that the modeling approach produces a valid approximation of the strain on the membrane.

However, the variation in these results need to discussed. The initial state of the test membrane is not pretension to remove any surface undulation. These undulations are clearly visible in Figure 16. The fact that strain Gauge 3 is located near these creases explains the deviation of the strain gauge measurement and predicted strain. Given the nature of the membrane material, it is unlikely the initial state of stress during installation is fully defined. This issue is aptly highlighted in the paper by Zhao et al. [11] where the definitions of the boundary conditions are required. As a result, one can only reasonably expect a definition of the change in the state of stress or strain unless the initial state of stress or strain is defined upon installation. In this regard, the most important benefit of this non-contact approach its ability to define the change in the state of stress of the entire membrane. The von Mises stress plot in Figure 21 identifies any region where the stress may be elevated by the formation of the scum-berg simulated with the addition of the spacers beneath the membrane. In practice, this will provide important information for the management of the membrane structure.

## 4. Determination of Strain Field from Photogrammetry on Realistic Scale Membrane Structure

### 4.1. Obtaining Geometry Using UAV and Photogrammetry

Figure 10 shows the capability of our UAV-aided photogrammetry in defining the geometry of a large floating cover at the Melbourne Water Waste Treatment Plant in Werribee measuring 470 m × 170 m. Given that the anaerobic processes occurring beneath the floating cover can lead to its deformation over time, the work presented in this section will demonstrate the ability to integrate the changing profile of the membrane structure as measured by the UAV into a finite element analyses methodology. This will allow determination of the change in the state of stress/strain arising from the change in the state of deformation of the floating cover.

**Figure 24:**
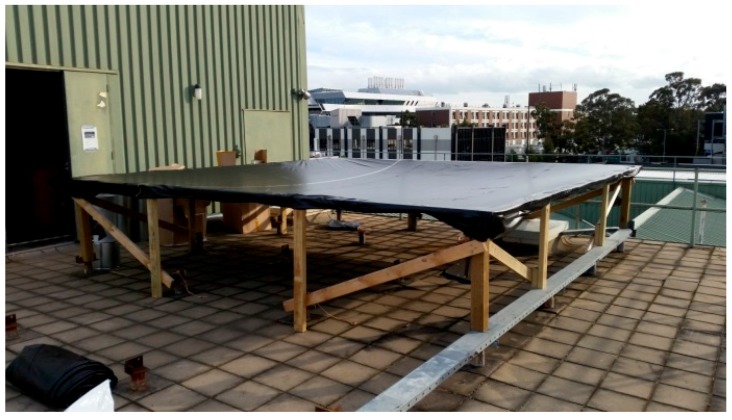
Photograph of the rooftop membrane.

This investigation was conducted on a test membrane measuring approximately 4.6 m × 4.6 m installed on the rooftop testing platform, as shown in Figure 24. The test membrane used is 0.5 mm thick PVC material. It was fastened to a rigid wooden frame using a series of self-tapping screws and washers. Once mounted, the membrane sagged in the middle due to its self-weight of approximately 12 kg.

**Figure 25:**
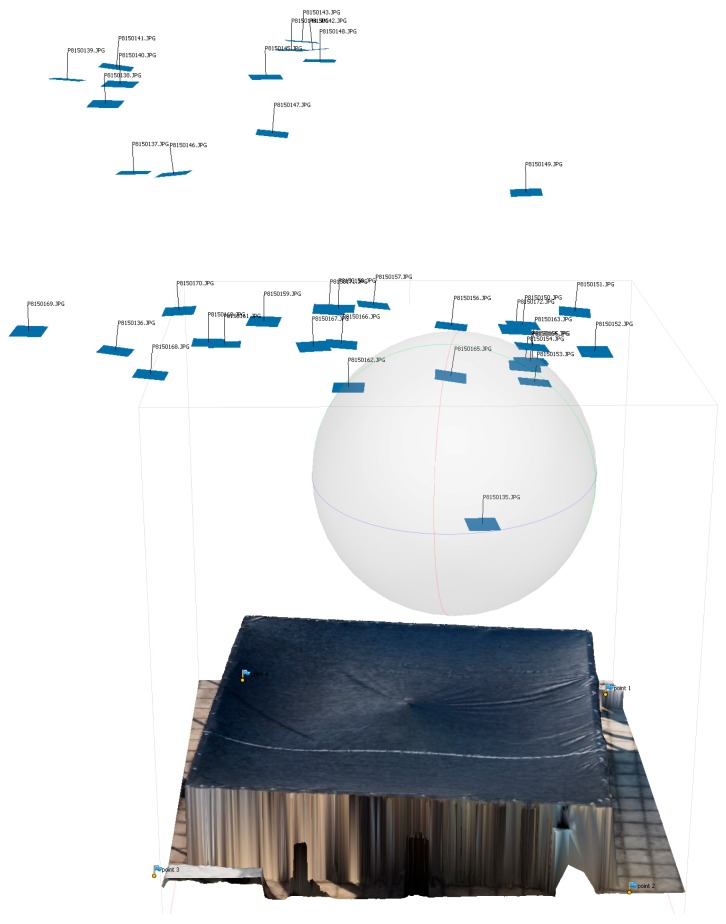
Location of photographs taken by UAV relative to rooftop structure.

**Figure 26:**
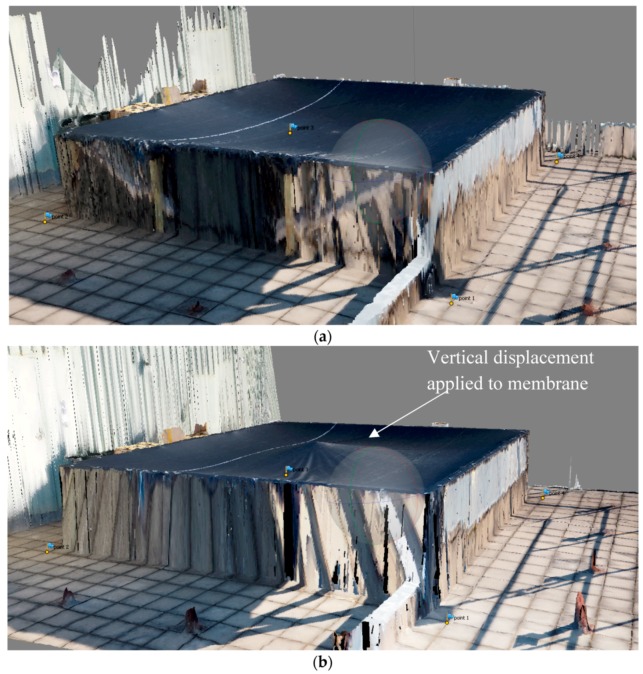
(**a**) 3D reconstruction of the rooftop membrane with edge supports (“initial” state); and (**b**) 3D reconstruction with the application of a vertical displacement in the middle of the membrane (“deformed” state).

**Figure 27:**
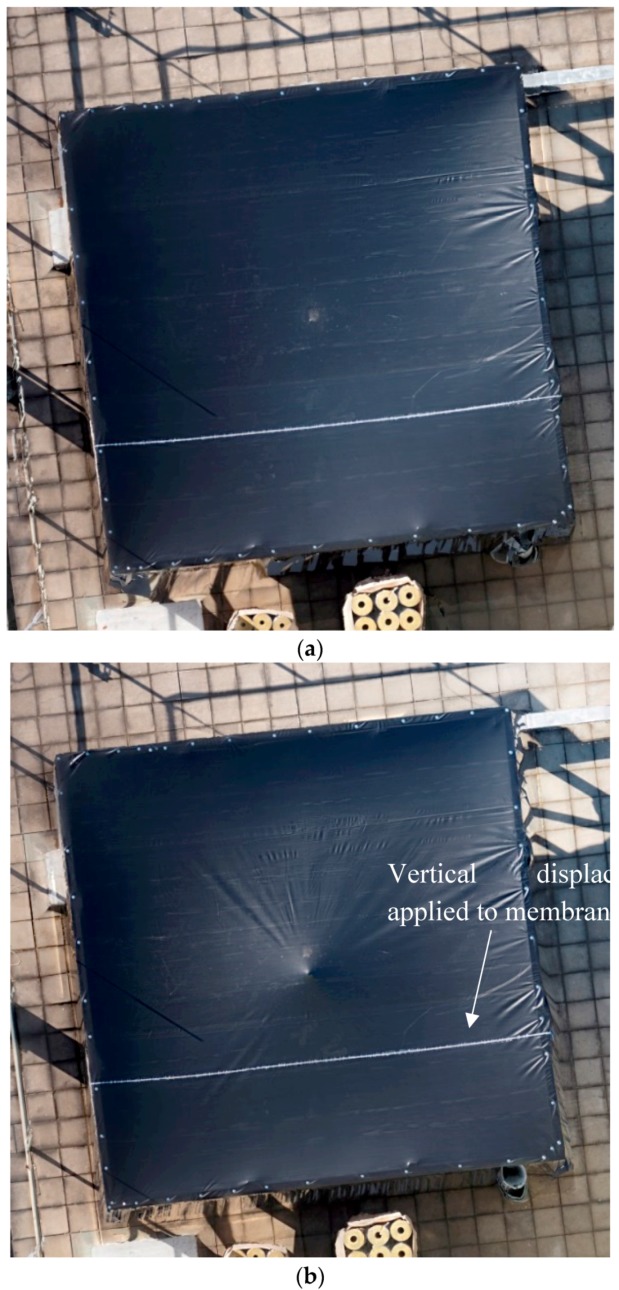
Orthophoto with two different loading conditions: (**a**) “initial” state; and (**b**) “deformed” state.

The membrane was scanned by the UAV in its “initial” state when it was only supported on the edges. The membrane was then scanned again in its “deformed” state where a tripod support was placed near the center which displaced the membrane approximately 40 mm (above the test frame) vertically to simulate the up-lift cause by the scum-berg. For each condition approximately 30 photos of the membrane structure were taken by the UAV. These were taken at various angles at 6 m above the rooftop over a period of 2 m. The UAV scans were conducted at near no-wind conditions. The recorded photos were post processed to reconstruct the membrane in 3D. Figure 25 shows the location of the pictures taken by the UAV over the membrane. The number of images taken and the overlap of each image was greater than nine. These parameters were determined from the results presented in Table 1. The flight parameters used to map the “initial” and “deformed” membrane are shown in Table 4. The “initial” and “deformed” reconstructions were aligned in 3D space by picking four fixed points outside of the membrane on the ground and matching them. A successful reconstruction was achieved despite having a “featureless” membrane resulting in Figure 26a,b. Figure 26a shows the re-construction of the membrane at its “initial” state, while the “deformed” state is shown in Figure 26b. The orthophoto of the test membrane at its “initial” state is shown in Figure 27a. Figure 27b shows the orthophoto of the test membrane in its “deformed” state.

**Figure 28:**
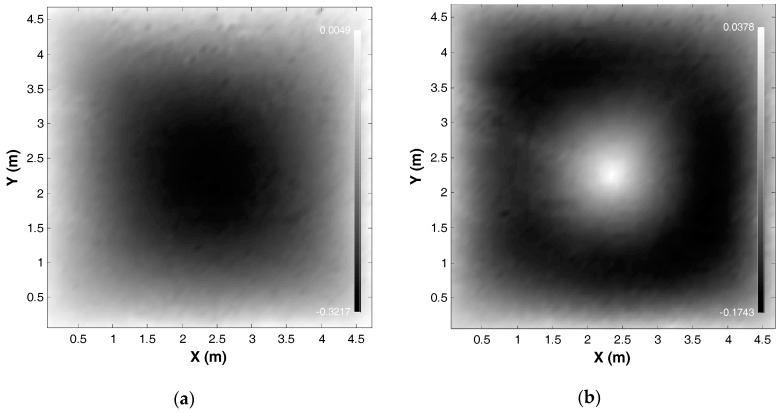
Elevation map: (**a**) “initial” state; and (**b**) “deformed” state.

The images of the test structure show that the displacement of the membrane is dominated by changes in out-of-plane direction and the in-plane displacements are minimal. This is expected as the loading imposed resulted from a vertical displacement applied to the membrane. To this end, the focus will only be on the out-of-plane displacement. In future work, the effects of inclusion of in-plane displacements with further image processing on the accuracy will be conducted. Figure 28 shows the elevation map for both conditions after the alignment process and referencing one corner to be the origin. The elevation maps of the “initial” and the “deformed” state of the test membrane are shown in Figure 28a,b, respectively.

### 4.2. Finite Element Model of the Test Membrane

A finite element model of the test membrane is prepared using commercially available Femap. A grid of 92 × 93 (8556 total) rectangular plate elements were used to represent the membrane. The test membrane geometry in its “initial” state was imported from the photogrammetry model captured by the UAV (Figure 27a). The geometry was then cropped to only feature the membrane and aligned to match the XY axis in the finite element model. After cropping the planar dimension, the membrane is 4.59 m × 4.61 m. From this point on, the procedure to integrate the photogrammetric output into the finite element model was identical to that used for the laboratory membrane study.

Fixed supports were defined on the membrane’s edge to emulate the wooden supports at the perimeter of the membrane. Load was applied to the membrane using the same procedure described for the laboratory study (Section 3). In this case, the “initial” state (S0) will be the membrane which is only edge supported and “deformed” state (S1) will be the membrane which is also supported by a tripod in the middle.

**Figure 29:**
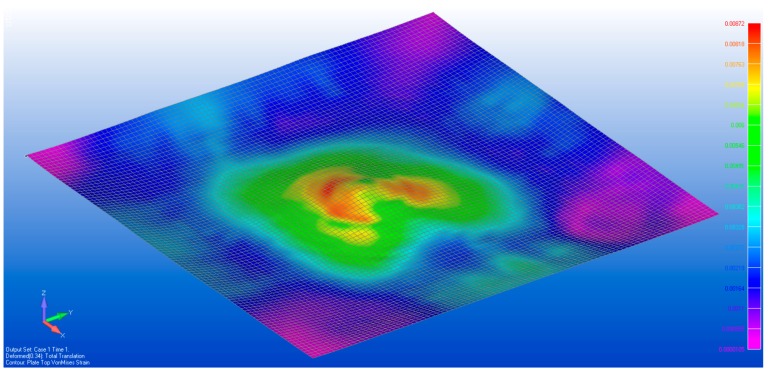
The von Mises strain caused by tripod support.

**Figure 30:**
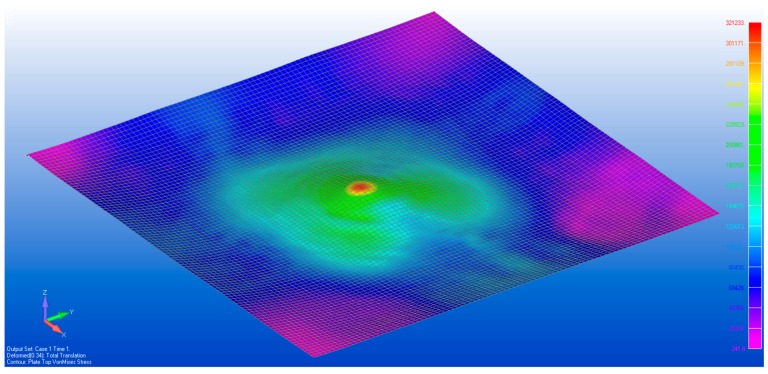
The von Mises stress caused by tripod support.

Figure 29 and Figure 30 show the vonMises equivalent strains and stress of the membrane when it is deformed from state S0 to S1, respectively. The largest change in the state of stress occurs around the location where the vertical displacements were enforced.

The above findings show the potential of a new structural health monitoring strategy for large membrane structures by integrating the elevation data provided by UAV into a representative finite element models. The scanning of a large area (1881 m^2^) took approximately 4 min. This provides an efficient means towards the development of a robust non-contact strain measurement strategy for large membrane. It must be emphasized that the current work is capable only of determining the change in the state of stress resulting from the deformation of the membrane. An adequate description of the boundary stresses/strains will be required if the absolute strain of the membrane is required.

## 5. Conclusions

A potential SHM system has been presented specifically for monitoring of membrane structures that undergo large deformations. A laboratory study was conducted where the aim was to make quantitative strain measurement using 3D scanning technology. The laboratory specimen zero stress shape was known and strain gauges were bonded to the specimen such that quantitative comparison could be made between this direct measurement and model prediction from 3D scanned geometry. This experiment demonstrated that the strain readings could quantitatively distinguish between the numbers of spacers used to load the membrane, confirming the validity of the model. The test was repeated on a much larger rooftop membrane structure, which confirms that the strain measurement can also be undertaken using UAV 3D scanning technology and on realistic scales. A key benefit of computing the stresses and strains through non-contact means is the ability to measure the entire membrane. This makes the non-contact approach an attractive option compared to conventional means, which can only measure a relatively small number of points. Furthermore, in the context of the floating cover, permanently attached sensors will degrade and weather over the cover’s expected life (over 20 years).

## Figures and Tables

**Table 1:** Average error (adapted from Chiu et al. [14]).

Flight Path	Flight Characteristics	Error (m)
#1	14.294 m altitude 5 m/s flight speed Photo captured every 3 m	2.09
#2	10.810 m altitude 3 m/s flight speed 50% overlap commanded.	0.95
#3	11.266 m altitude 2 m/s flight speed 70% overlap commanded.	0.46

**Table 2:** Strain readings from the strain gauges.

	Strain Gauge (Micro-Strain)			
# Spacers	1	2	3	4
0	0	0	0	0
1	1124	2208	596	2837
2	4358	4650	1726	6025
3	10,950	9942	6105	13,150

**Table 3:** Strain readings from the FE model.

	FE Strain at Gauge Location (Micro-Strain)			
# Spacers	1	2	3	4
0	0	0	0	0
1	1046	1475	1342	968
2	4465	4367	6032	4722
3	8915	10,790	10,830	8747

**Table 4:** Flight parameters.

	With Tripod	Without Tripod
Flight altitude (m)	7.158	7.745
Number of images	38	27
